# Shining a Light on BP-3 Exposure: Sunscreen Chemical Measured in U.S. Population

**DOI:** 10.1289/ehp.116-a306a

**Published:** 2008-07

**Authors:** John Tibbetts

Sunscreens provide important protection against sunburn and squamous cell cancer, particularly for individuals who work outdoors or in situations where sun exposure is unavoidable. The widespread use of the common sunscreen agent benzophenone-3 (BP-3) and its detection in the environment suggest the need for more information about the extent of human exposure. Results of a new study now provide the first nationally representative data on BP-3 exposure among the U.S. population **[*EHP* 116:893–897; Calafat et al.]**.

BP-3 is used in personal care products to absorb and dissipate ultraviolet (UV) radiation. It is also used as a UV stabilizer in plastic surface coatings to prevent polymer or food photodegradation and has been approved by the Food and Drug Administration as an indirect food additive. Although BP-3 exposure has not been linked to adverse health effects in humans, results of animal studies by the National Toxicology Program have shown effects in liver, kidney, and reproductive organs, and studies by other groups have shown endocrine-disrupting effects.

Using data from the National Health and Nutrition Examination Survey (NHANES) 2003–2004 conducted by the Centers for Disease Control and Prevention, the current research team analyzed 2,517 urine samples from three major racial/ethnic groups: non-Hispanic black, non-Hispanic white, and Mexican American. NHANES includes household interviews, medical histories, standardized physical examinations, and a collection of biologic specimens that can be used to assess exposure to environmental chemicals, as in the current study.

The scientists detected BP-3 in 96.8% of the urine samples, with a mean concentration of 22.9 μg/L and a concentration of 1,040 μg/L in the 95th percentile. The high level of detection likely resulted from routine use of personal care products such as sunscreen, moisturizers, lipstick, and hairspray.

Results of the current study suggest that females and non-Hispanic whites were the most highly exposed of all the demographic groups studied. Mean concentrations of BP-3 were significantly higher for females than for males, regardless of age, probably because women and girls use more sunscreen and other personal care products than men and boys do. At the 95th percentile of exposure, adult females had BP-3 concentrations 3.5 times greater than those of adult males.

Mean concentrations also differed significantly among the different racial/ethnic groups. Non-Hispanic whites were 6.8 times more likely and Mexican Americans were 4 times more likely to have BP-3 concentrations above the 95th percentile compared with non-Hispanic blacks. These differences likely result from increased use of sunscreens by people with lighter skin pigmentation.

According to the authors, the NHANES 2003–2004 data can be used to establish a nationally representative baseline assessment of exposure. Moreover, the data could aid risk assessments for BP-3 exposure if future toxicologic or epidemiologic studies suggest the need for such research, and may encourage further research to determine the potential public health impact of exposure at the levels reported.

## Figures and Tables

**Figure:**
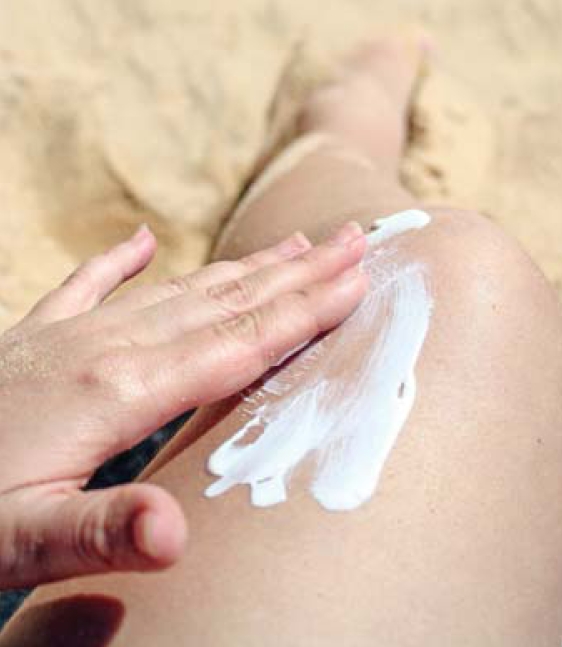
In an NHANES sample, women and light-skinned individuals had the highest concentrations of the sunscreen agent BP-3

